# Genomic breeding value estimation using nonparametric additive regression models

**DOI:** 10.1186/1297-9686-41-20

**Published:** 2009-01-27

**Authors:** Jörn Bennewitz, Trygve Solberg, Theo Meuwissen

**Affiliations:** 1 Department of Animal and Aquacultural Sciences, Norwegian University of Life Sciences, Box 1432, Ås, Norway; 2 Institute of Animal Breeding and Husbandry, Christian-Albrechts-University of Kiel, 24098 Kiel, Germany

## Abstract

Genomic selection refers to the use of genomewide dense markers for breeding value estimation and subsequently for selection. The main challenge of genomic breeding value estimation is the estimation of many effects from a limited number of observations. Bayesian methods have been proposed to successfully cope with these challenges. As an alternative class of models, non- and semiparametric models were recently introduced. The present study investigated the ability of nonparametric additive regression models to predict genomic breeding values. The genotypes were modelled for each marker or pair of flanking markers (*i.e*. the predictors) separately. The nonparametric functions for the predictors were estimated simultaneously using additive model theory, applying a binomial kernel. The optimal degree of smoothing was determined by bootstrapping. A mutation-drift-balance simulation was carried out. The breeding values of the last generation (genotyped) was predicted using data from the next last generation (genotyped and phenotyped). The results show moderate to high accuracies of the predicted breeding values. A determination of predictor specific degree of smoothing increased the accuracy.

## Introduction

Genomic selection refers to the use of genomewide dense marker genotypes for breeding value estimation and subsequently for selection. Genomic breeding value estimation relies on linkage disequilibrium (LD) between genetic markers and QTL and needs genomewide and dense marker data. The main challenge is the estimation of many effects from a limited number of observations. To cope with this problem, Meuwissen *et al*. [1] proposed Bayesian methods that used informative priors. Meuwissen *et al*. [1] and Solberg *et al*. [2] showed by means of simulations that these methods are able to estimate genomic breeding values with a remarkably high accuracy, even for individuals without own phenotypic observations. This offers the opportunity to speed up genetic gain by reducing the need for progeny testing [3].

Gianola *et al*. [4] argued that the assumptions made in the Bayesian models of Meuwissen *et al*. [1] are rather strong (*e.g*. the priors are very informative) and introduced nonparametric and semiparametric models, which make fewer assumptions. Two ways of modelling the genotypic data are presented by these authors. The first models all genotypes of an individual across the genome simultaneously; see eq. (1) of Gianola *et al*. [4]. Subsequently, the non- or semiparametric estimate includes additive genetic effects as well as dominance and epistasis. From this total genomic value, an additive breeding value can be extracted by performing linear approximations as shown in eq. (8) of Gianola *et al*. [4]. In the second way of modelling, the genotypes are modelled for each locus separately, see eq. (7) of Gianola *et al*. [4]. The authors [4] suggest estimating the nonparametric functions of the genotypes of a certain locus by applying additive model theory [5]. This way of modelling ignores epistatic effects.

The total genomic value of an individual is of interest in many cases, favouring the first way of modelling the genotypic data in Gianola *et al*. [4]. For example, one might think of classifying individuals with respect to their liability to a certain disease. In most livestock selection schemes, however, the breeding values, defined as the sum of the additive effects [6], are in general the most important. Following this, the second way of modelling the genotypic data in Gianola *et al*. [4], as described above, seems to be an interesting option, because it yields directly the additive effects, if the genotypes are modelled appropriately, and no extra computational step for the linear approximation is needed.

The aim of the present study was to investigate the ability of kernel regression using additive models to estimate genomic breeding values. In particular, the modelling of the genotypic data is shown and a method for the optimal selection of model parameters is presented. Using simulations, the accuracy of predicted breeding values from non-phenotyped animals were evaluated. The results were compared to those obtained from the BLUP method for genomic breeding value estimation.

## Methods

### Nonparametric kernel regression using additive models

Assume that *n *individuals (*i *= 1, ..., *n*) are genotyped at *N *single nucleotide polymorphisms (SNPs) (*j *= 1, ..., *N*). Biallelic SNP are considered. In this case, *q *= 2 different alleles are possible at a SNP (*l *= 1, *q*). An allele is coded as 0 or 1 and is denoted by *x*. The individuals are diploid, thus they have two chromosomes (*k *= 1, 2). Further, the individuals are phenotyped for a heritable quantitative trait. The phenotypes are denoted by *y *and are free of systematic errors. In the additive allelic model, the phenotype of an individual is represented as

(1) $$y_{i}=\sum_{j=1}^{N}\sum_{k=1}^{2}g_{j}(x_{ijk})+e_{i},$$

where *x*_*ijk *_is the *k*th allele of individual *i *at marker locus *j *and *g*_*j*_(*x*_*ijk*_) is the function value of the *k*th allele at this locus. *e*_*i *_is a normally distributed random residual. The conditional expectation function is

(2a) *g*_*j*_(*x*_*ijk*_) = *E*(*y*_*i *_|*x*_*ijk*_),

The conditional expectation function for any locus *j *with its alleles *x*_*jl *_can be written in terms of densities [7]

(2b) $$g_{j}(x_{jl})=\frac{\int yp(x_{jl},y)dy}{p(x_{jl})},$$

where *p*(*x*_*jl*_) is the density of *x*_*jl *_and can be estimated using a kernel smoother as

(3) $$\hat{p}(x_{jl})=\frac{1}{2n\lambda^{N}}\sum_{i=1}^{n}\sum_{k=1}^{2}K\left(\frac{x_{ik}-x_{jl}}{\lambda }\right),$$

where *K *denotes for the kernel and *λ *for a smoothing parameter. In (3), *x*_*jl *_is the point at which the density is estimated, this is termed the focal point [7]. The joint density of *x*_*jl *_and *y *at point (*x*_*jl *_,*y*) is estimated as

(4) $$\hat{p}(x_{jl},y)=\frac{1}{2n\lambda^{N}}\sum_{i=1}^{n}\sum_{k=1}^{2}K\left(\frac{x_{ik}-x_{jl}}{\lambda }\right)K\left(\frac{y_{i}-y}{\lambda }\right).$$

Now, it can be shown [*e.g*. [4,8]] that substituting (3) and (4) in (2b) results in the Nadaraya-Watson kernel regression estimator [9,10] for the conditional expectation function *g*_*j*_(*x*_*jl*_)

$${\hat{g}}_{j}(x_{jl})=\frac{\sum_{i=1}^{n}\sum_{k=1}^{2}K((x_{ik}-x_{jl})/\lambda )y_{i}}{\sum_{i=1}^{n}\sum_{k=1}^{2}K((x_{ik}-x_{jl})/\lambda )}.$$

The additive haplotype model is similar to the allelic model except that haplotypes, formed by pairs of flanked markers, are considered instead of single allelic marker effects. Consequently, the outlines shown above hold, if it is assumed that *x*_*ijk *_is the *k*th haplotype at chromosome segment *j *of individual *i *and the first summation in (1) is over *N *segments. The coding of the haplotypes is done so that *x *can take *q *= 4 different values, *i.e*. 1-1, 1-0, 0-1, or 0-0. Similarly, the functions of the segments are estimated using the Nadaraya-Watson regression estimator. In the following no distinction is made between the allelic and the haplotype model, unless stated. The loci and segments are both denoted as predictors and the alleles and haplotypes both as levels of the predictors, or short, as levels.

The *x*_*ijk *_are discrete with only *q *= 2 (*q *= 4) different values in the allelic (haplotype) model, see above. Therefore we choose the binomial kernel of Aitchison and Aitken [11]. Using this kernel, for each focal *x*_*jl *_and each observed *x*_*ij *_the number of disagreements *d *is estimated. In the allelic model *d *takes values of 0 (*e.g. x*_*jl *_is 0 and *x*_*ij *_is 0) or 1 (*e.g. x*_*jl *_is 0 and *x*_*ij *_is 1), and in the haplotype model values of 0 (*e.g. x*_*jl *_is 1-1 and *x*_*ij *_is 1-1), 1 (*e.g. x*_*jl *_is 1-1 and *x*_*ij *_is 1-0 or 0-1) or 2 (*e.g. x*_*jl *_is 1-1 and *x*_*ij *_is 0-0). Using this definition of *d*, the binomial kernel *K *is

$$K(x_{jl},x_{ij},\lambda )=\lambda^{q-d(x_{jl},x_{ij})}{\left(1-\lambda \right)}^{d(x_{jl},x_{ij})},$$

where *λ *is the smoothing parameter with $\frac{1}{2}$ ≤ *λ *≤ 1 [11]. The Nadaraya-Watson regression applying the binomial kernel for the estimation of the functions is

(5) $${\hat{g}}_{j}(x_{jl})=\frac{\sum_{i=1}^{n}\sum_{k=1}^{2}\lambda^{q-d(x_{jl},x_{ijk})}{\left(1-\lambda \right)}^{d(x_{jl},x_{ijk})}y_{i}}{\sum_{i=1}^{n}\sum_{k=1}^{2}\lambda^{q-d(x_{jl},x_{ijk})}{\left(1-\lambda \right)}^{d(x_{jl},x_{ijk})}}.$$

Extending (2a) to account for multiple predictors, the conditional expectation function can be written as

(6) $$g_{j}(x_{ijk})=E\left[(y_{i}-\sum_{\begin{array}{l} j^{\prime }=1\\ j^{\prime }\neq j \end{array}}^{N}\sum_{k=1}^{2}g_{j^{\prime }}(x_{ij^{\prime }k})|x_{ijk})\right].$$

Assuming additivity of the predictors, this leads to the following iterative backfitting algorithm [12,5] for computing the functions.

1. ***j *= 1, ..., *N*; Initialise **${\hat{g}}_{j}$(*x*_*jl*_).

2. ***j *= 1, ..., *N***; ${\hat{g}}_{j}$(*x*_*jl*_) = NWR($y_{i}^{*}$ | (*x*_*ijk*_). **Centre **${\hat{g}}_{j}$(*x*_*jl*_).

3. **Repeat step 2 until convergence is reached**.

In step one the nonparametric function values are initialised with some small numbers. Step two comprises the application of the Nadaraya-Watson regression (denoted by NWR) in the form described in (5), but using ($y_{i}^{*}$ | *x*_*ijk*_) instead of *y*_*i*_. The term ($y_{i}^{*}$ | *x*_*ijk*_) is called the partial residual and denotes for the phenotypes corrected for every predictor except for the level *k *of individual *i *at predictor *j*. The collinearities result in a non-uniqueness of the estimates [5]. Therefore, ${\hat{g}}_{j}$(*x*_*jl*_) are centred in the second step by subtracting the mean of fitted function values to the 2*n *chromosomes at the predictor *j*. This centring ensures that the overall mean of the fitted function values is zero at every cycle of the backfitting and the algorithm converges to one possible solution [5]. It might be noted that the backfitting algorithm is very similar to the Gauss-Seidel algorithm, further details can be found in [5].

### Choosing the smoothing parameter *λ*

In applying kernel regression, one key question is which value for the smoothing parameter *λ *should be used. As stated above, when a binomial kernel is applied, the lower and upper bound of *λ *is 0.5 and 1, respectively. When *λ *= 1 the whole weight of *K*(*x*_*jl*_, *x*_*ij*_, *λ*) is concentrated at *x*_*ij *_= *x*_*jl *_and $\hat{p}$(*x*_*jl*_) in (3) is just the proportion of cases *x*_*jl *_was observed in the sample. On the contrary, when *λ *= 0.5, the degree of smoothing is at maximum and *K*(*x*_*jl*_, *x*_*ij*_, *λ*) gives the same weight to each of the *x*_*jl *_[11,7]. One way of selecting an appropriate *λ *is to apply bootstrapping as follows [13]. Assume a number of *B *bootstrap samples (*b *= 1, ..., *B*). In each *b*, the data points are split into two sets. The first set, denoted as the estimation set, is formed by the entire bootstrap sample and the second, denoted as the test set, is formed by the individuals not found in the corresponding bootstrap sample. Since a bootstrap sample is generated by drawing *n *observations out of the original pool of *n *observations with replacement [13], the probability of any given progeny being chosen after *n *drawings is [1-[1-1/*n*]^*n*^] ≈ 0.632 and the probability not being chosen, and consequently forming the test set, is [1-1/*n*]^*n *^≈ e^-1 ^≈ 0.368. For each individual an indicator variable *k*_*i *_is introduced, this is 1 if the individual is present in the test set of the corresponding bootstrap sample *b*, and 0 otherwise (*k*_*ib *_= 1 and *k*_*ib *_= 0, respectively). For a grid of *λ *and each bootstrap sample *b*, the functions of each predictor *j *are estimated as described above using the corresponding estimation set of each *b*. This results in *B *different ${\hat{g}}_{\lambda ,j}^{b}$. The average residual sums of squares of each individual is calculated as

(7a) $$aveRSS_{\lambda }^{i}=\frac{1}{\sum_{b=1}^{B}k_{ib}}\sum_{b=1}^{B}k_{ib}*{\left(y_{i}-\sum_{j=1}^{N}\sum_{k=1}^{2}{\hat{g}}_{\lambda ,j}^{b}(x_{ijk})\right)}^{2}.$$

This means that only those bootstrap samples are considered where the corresponding individual *i *was not in the estimation set, but in the test set. Averaging over all individuals yields

(7b) $$aveRSS_{\lambda }=\frac{1}{n}\sum_{i=1}^{n}aveRSS_{\lambda }^{i}.$$

Note that the subscript *i *denotes for the individual. The *λ*, which produced the smallest *aveRSS*, can be chosen to analyse the original sample. This method is termed the equal lambda method (ELM) in the following, because the *λ *takes the same value for each predictor.

Different *λ *might be optimal for different predictors and a predictor specific determination of *λ *is desirable. In principle, the bootstrap strategy can be expanded accordingly. However, this would need *B *times *N *times the number of *λ *in the grid calculations, which is computationally not feasible. Additionally, the constellation, which results in the smallest *aveRSS *might be difficult to find. In previous analysis we investigated the optimal degree of smoothing for predictors taking the knowledge of the simulated QTL into account. The degree of smoothing was less for predictors in LD with a QTL compared to predictors not in LD with a QTL. Additionally, predictors that showed a similar variance of their function values, also showed a similar optimal *λ*. This lead to the following algorithm for the group-wise predictor specific *λ *determination, subsequently named unequal lambda method (ULM).

1. Determine one *λ *valid for all predictors using ELM.

2. Estimate the variance of the *q *function values for each predictor (*q *= 2 in the allelic and *q *= 4 in the haplotype model, see above).

3. Select those *m *(*e.g. m *= 5) predictors which show the highest variance and determine an optimal *λ *for them using bootstrapping, but letting the lower bound of *λ *be as determined in ELM. The *λ *for the remaining predictors are fixed at the determined value from ELM.

4. Repeat step 3 for the next set of *m *predictors, which show the next highest variance. Here, keep *λ *for the remaining predictors fixed at their determined value, *i.e*. from ELM for predictors with a lower variance, and from step (3) otherwise.

5. Repeat step 4 until all predictors are passed.

Finally, the original sample is analysed with the group-wise predictor specific *λ*.

### BLUP method for genomic breeding value estimation

The BLUP model of Meuwissen *et al*. [1] can be applied in an allelic model or in a haplotype model. For simplicity only the allelic BLUP model will be considered in the following. In Meuwissen *et al*. [1] it is assumed that the variance of a marker effect is $\sigma_{a}^{2}$/(2*N*), with $\sigma_{a}^{2}$ being the additive genetic variance. Note that each marker affects the phenotype two times, via the paternal and the maternal allele, hence the *2N *in the denominator. If the unequal gene frequencies at the markers are taken into account, the variance of a marker effect becomes $\sigma_{a}^{2}$/(4*N*$\bar{H}$), with $\bar{H}$ being the average heterozygosity across markers. The derivation is given in the Appendix 1, and can also be found in Habier *et al*. [14] using a different approach. If $\bar{H}$ equals 0.5 (*i.e*. the allele frequency at every marker is 0.5), the expression reduces to $\sigma_{a}^{2}$/(2*N*).

### Simulations

In order to test the ability of the additive nonparametric regression models to predict reliable breeding values, and to compare the results from those obtained from BLUP, a simulation study was conducted. The simulations were performed as described by Solberg *et al*. [2]. Briefly, a population was simulated over 1000 generations with mutations and random selection and mating with an effective population size of 100. Ten chromosomes each of 100 cM length and each with 100 potential QTL evenly distributed over the chromosome were generated. The number of segregating QTL depended on the mutation rate at the QTL, which was assumed to be 2.5 × 10^-5 ^[2]. For each mutation at the QTL an additive effect was sampled from the gamma distribution with a shape and a scale parameter of 1.66 and 0.4, respectively [15]. This implied that many QTL had small and only few had large effects. QTL effects were sampled such that they had equal probability of positive or negative effects. QTL effects were simulated to be additive. The marker density was 1 cM, 0.5 cM or 0.25 cM. The mutation rate at the markers was assumed to be 2.5 × 10^-3 ^[2]. Markers showed in general multiple alleles. In order to reflect SNP markers, they were converted to biallelic markers by assuming that only one of the mutations was visible as described by Solberg *et al*. [2]. The proportion of segregating SNPs (segregating QTL) was around 98% (5–6%) of the number of simulated markers (QTL) at generation 1000. In generation 1001, the number of animals was increased to 1000 by factorial mating. The LD of pairs of segregating markers was estimated as *r*^2 ^value in generation 1001. The average *r*^2 ^of two adjacent segregating markers was 0.158, 0.222, and 0.295 for the marker density 1 cM, 0.5 cM and 0.25 cM, respectively [2]. The animals in generation 1001 produced 1000 offspring for generation 1002 by random mating. Animals in generation 1001 and 1002 were genotyped at the SNP markers and animals in generation 1001 were also phenotyped. The phenotypes were the sum of their simulated breeding value and a random deviation e (e ~ N(0, $\sigma_{e}^{2}$)). $\sigma_{e}^{2}$ was chosen such that the heritability of the trait was h^2 ^= 0.25 or h^2 ^= 0.5. For the haplotype model, the simulated haplotypes were used (no extra haplotype determination was performed). The number of replicates was 10 for each marker density and each h^2^.

In the additive nonparametric regression, the functions were estimated using the data from the generation 1001. These were used to predict the breeding values (*EBV*) of the generation 1002 as

$$EBV_{i}=\sum_{j=1}^{N}\sum_{k=1}^{2}{\hat{g}}_{j}(x_{ijk}).$$

The smoothing parameter *λ *was varied as *λ *= 0.5, 0.525, .... A total of *B *= 50 bootstrap samples were generated. For ULM, the groups size for the group-wise predictor specific *λ *determination was *m *= 5, 10 and 20 for a marker density of 1 cM, 0.5 cM and 0.25 cM, respectively. The convergence criterion to exit the backfitting algorithm was an average change of the function values of two consecutive iterations below 2.5 * 10^-5^. A relaxation factor [*e.g*. [16]] of 0.7 was included. Additionally, generation 1001 was analysed using the BLUP model described above, assuming the variance of the effects of each marker is $\sigma_{a}^{2}$/(4*N*$\bar{H}$) and using the simulated variance components. The BLUP system of equations was solved iteratively by applying the Gauss-Seidel algorithm [*e.g*. [16]]. The same convergence criterion as for the nonparametric additive model was used. Also these estimates were used to predict the breeding values of generation 1002.

The correlation between the true breeding value and the *EBV *of the individuals in generation 1002 as well as the regression coefficient of the *TBV *on the *EBV *was estimated, which served as empirical measures of the ability of the methods to predict accurate and unbiased breeding values of individuals without own phenotypic observations [1]. Unbiased means here *E*(*TBV*|*EBV*) = *EBV*, and a regression coefficient below one (above one) indicates that the *EBV *vary too much (too little). Unbiased EBV are important if selection has to be carried out from multiple generations using estimated marker effects in one generation. Assume selection will be done across two-year classes, where the marker effects are estimated in the older year class only. Further assume that the younger year class is in general superior (*i.e*. has a higher population mean) due to selection response. If the *EBV *vary too much (too little) then too many animals will be selected from the older (younger) year class.

## Results

The results are shown in Tables 1 and 2. Summarized over all genetic configurations analyzed, the accuracies of *EBVs *obtained from ULM were highest. However, these were also most biased, as indicated by the in general lower regression coefficients. The accuracies from ELM and BLUP were very similar.

**Table 1:** Results from the prediction of the breeding values of the last generation using data from the next last generation as a function of the marker density

Method	Model		Marker density		
			1 cM	0.5 cM	0.25 cM
ELM	allelic	*r*_TBV,EBV_^a^	0.531 (0.058)	0.552 (0.043)	0.629 (0.039)
		*b*_TBV,EBV_^b^	1.017 (0.139)	0.848 (0.106)	0.722 (0.075)
	haplotype	*r*_TBV,EBV_	0.534 (0.055)	0.561 (0.044)	0.626 (0.033)
		*b*_TBV,EBV_	0.829 (0.066)	0.778 (0.049)	0.679 (0.029)
ULM	allelic	*r*_TBV,EBV_	0.560 (0.078)	0.617 (0.035)	0.641 (0.036)
		*b*_TBV,EBV_	0.754 (0.106)	0.720 (0.092)	0.626 (0.070)
	haplotype	*r*_TBV,EBV_	0.575 (0.076)	0.614 (0.040)	0.637 (0.035)
		*b*_TBV,EBV_	0.711(0.071)	0.610 (0.041)	0.567 (0.029)
BLUP	allelic	*r*_TBV,EBV_	0.532 (0.061)	0.549 (0.042)	0.622 (0.042)
		*b*_TBV,EBV_	1.143 (0.098)	1.178 (0.110)	1.376 (0.086)

The heritability was 0.25. Average from 10 replicates. ELM and ULM denotes for equal lambda and unequal lambda method, respectively.

^a ^Correlation between true and estimated breeding value; standard deviations are in parenthesis

^b ^Regression of true on estimated breeding value; standard deviations are in parenthesis

**Table 2:** Results from the prediction of the breeding values of the last generation using data from the next last generation as a function of the marker density

Method	Model		Marker density		
			1 cM	0.5 cM	0.25 Cm
ELM	allelic	*r*_TBV,EBV_^a^	0.642 (0.074)	0.670 (0.029)	0.783 (0.025)
		*b*_TBV,EBV_^b^	1.101 (0.125)	1.002 (0.073)	0.968 (0.023)
	haplotype	*r*_TBV,EBV_	0.645 (0.064)	0.671 (0.028)	0.785 (0.023)
		*b*_TBV,EBV_	1.024 (0.117)	0.982 (0.094)	0.921 (0.018)
ULM	allelic	*r*_TBV,EBV_	0.679 (0.091)	0.733 (0.029)	0.805 (0.018)
		*b*_TBV,EBV_	0.937 (0.102)	0.886 (0.074)	0.865 (0.024)
	haplotype	*r*_TBV,EBV_	0.692 (0.076)	0.747 (0.028)	0.810 (0.014)
		*b*_TBV,EBV_	0.898 (0.085)	0.851 (0.058)	0.883 (0.026)
BLUP	allelic	*r*_TBV,EBV_	0.641 (0.067)	0.667 (0.029)	0.773 (0.029)
		*b*_TBV,EBV_	1.070 (0.110)	1.147 (0.085)	1.219 (0.033)

The heritability was 0.5. Average from 10 replicates. ELM and ULM denotes for equal lambda and unequal lambda method, respectively.

^a ^Correlation between true and estimated breeding value; standard deviations are in parenthesis

^b ^Regression of true on estimated breeding value; standard deviations are in parenthesis

The impact of the heritability can be seen when comparing the results reported in Table 1 with those in Table 2. As expected, the accuracies of the *EBVs *were higher for a heritability of 0.5. Additionally, the *EBVs *were in general less biased for the higher heritability. This was most obvious for ULM. Increasing marker density led to higher accuracies of *EBVs *for all methods. With increasing marker density the regression coefficient of the true on the estimated breeding value decreased for ELM and ULM, resulting in general in an increased bias with increasing marker density. One exception is for ELM and a marker density of 1 cM, where the *EBVs *vary too little. Here, the bias decreased when moving to a marker density of 0.5 cM (see second row of Tables 1 and 2). In contrast, with increasing marker density the regression increased for BLUP.

The differences between the allelic and the haplotype model were small, regardless of the method used (Tables 1 and 2). The haplotype model produced slightly better results in low marker density situations, but with dense markers the accuracies from the allelic and the haplotype model were very similar. The same was reported for the BayesB method [17,2].

The computational demand was in an increasing order: BLUP, ELM and ULM. For example, one replicate with a marker density of 1 cM analysed with the allelic model took below one minute when using BLUP, around one hour for ELM and several hours for ULM. The reason is, that ELM and ULM included bootstrapping to determine the optimal *λ*. Naturally, the computation time would even be higher if the number of bootstrap samples (*B*) would be larger. It seems that *B *= 50 is at the lower bound when comparing with literature reports [13]. However, increasing *B *did not produce significantly different results (not shown), indicating that *B *= 50 was sufficient here. The time to reach convergence depended on *λ *and the marker density. With increasing *λ *and increasing marker density more iteration were needed until convergence was reached. For example, in general the number of iterations for *λ *= 0.6 was ~15 and for *λ *= 0.9 was ~50 for a marker density of 1 cM. The same figures for a marker density of 0.25 cM were ~20 and ~90, respectively.

**Figure 1:**
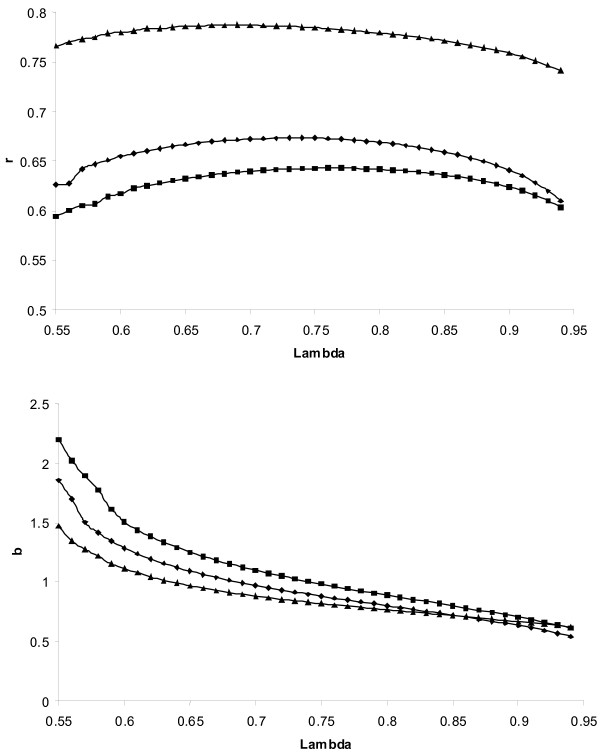
**Results from the allelic additive nonparametric regression**. Correlation (*r*) between the true and the estimated breeding values (top) and regression (*b*) of the true on the estimated breeding values (bottom) as a function of smoothing parameter (lambda) and the marker density. The same lambda was applied to all markers. The heritability was 0.5 and marker density was 1 cM (black square), 0.5 cM (black diamond), and 0.25 cM (black triangle), respectively. Average from 10 replicates.

**Figure 2:**
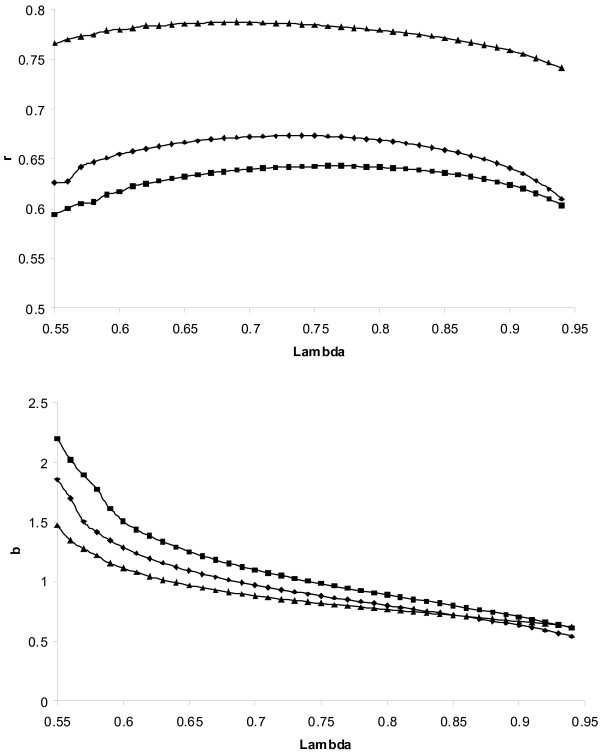
**Results from the haplotype additive nonparametric regression**. Correlation (*r*) between the true and the estimated breeding values (top) and regression (*b*) of the true on the estimated breeding values (bottom) as a function of smoothing parameter (lambda) and the marker density. The same lambda was applied to all chromosomal segments. The heritability was 0.5 and marker density was 1 cM (black square), 0.5 cM (black diamond), and 0.25 cM (black triangle), respectively. Average from 10 replicates.

Figure 1 and 2 showed that during the grid search for the optimal *λ*, the accuracy increased with increasing *λ *monotonically and decreased monotonically after the optimum *λ *was passed. Therefore, in order to speed up computations, the grid was started at the lower bound of *λ *and was ended when the *aveRSS *from (7a) and (7b) stopped decreasing, assuming that the optimal *λ *was reached or is not far away. The start at the lower bound was because convergence is reached fast if *λ *is small (see above). Additionally, if *aveRSS *failed to decrease due to some random sampling before the optimal *λ *was reached, this would result in an over-smoothing, and hence, the results would be conservative.

For ULM the numbers of predictors with a *λ *within a defined bin are shown in Tables 3 and 4. A higher marker density results in more predictors that are less smoothed, *i.e*. showing a *λ *closer to one. This is due to the higher number of predictors in LD with the QTL. Also, with an increased heritability more predictors are less smoothed (top and bottom of Tables 3 and 4). The grid search for finding the optimal *λ *is more powerful in high heritability situations, leading to this lesser degree of smoothing. Additionally, as for ELM, more smoothing is done in the haplotype model than in the allelic model. This can be seen in the higher number of predictors showing a *λ *> 0.9 in the allelic model (Table 3 and 4).

**Table 3:** Results from the unequal lambda method (ULM)

Heritability	Model	0.6 <*λ *< 0.7	0.7 ≤ *λ *< 0.8	0.8 ≤ *λ *< 0.9	0.9 ≤ *λ *< 1
0.25	allelic	976.5 (9.0)	2.0 (4.8)	4.5 (5.5)	17.0 (6.8)
	haplotype	973.0 (5.9)	3.5 (4.1)	5.0 (3.3)	8.5 (5.8)
0.5	allelic	0.0	972.2 (9.8)	3.9 (4.9)	23.8 (9.3)
	haplotype	968.0 (7.2)	0.5 (1.6)	9.0 (6.6)	12.5 (5.4)

Number of marker locus (allelic model) or chromosomal segments (haplotype model) showing a smoothing factor (*λ*) in the corresponding bin for a marker density of 1 cM. Average from 10 replicates. Standard deviations are in parenthesis.

**Table 4:** Results from the unequal lambda method (ULM)

Heritability	Model	0.6 <*λ *< 0.7	0.7 ≤ *λ *< 0.8	0.8 ≤ *λ *< 0.9	0.9 ≤ *λ *< 1
0.25	allelic	1961.0 (17.9)	1.0 (3.2)	6.0 (8.4)	32.0 (16.2)
	haplotype	1951.0 (13.7)	5.0 (5.3)	18.0 (13.9)	16.0 (8.4)
0.5	allelic	578.0 (933.9)	358.0 (937.2)	7.0 (9.5)	57.0 (17.7)
	haplotype	1940.0 (18.9)	10.0 (8.2)	23.0 (14.9)	17.0 (4.8)

Number of marker loci (allelic model) or chromosomal segments (haplotype model) showing a smoothing factor (*λ*) in the corresponding bin for a marker density of 0.5 cM. Average from 10 replicates. Standard deviations are in parenthesis.

## Discussion

As stated in the introduction, in genomic breeding value estimation we are faced with the problem of estimating many effects from a limited number of observations, and, additionally, many effects show collinearities due to the LD between the SNPs. The BLUP model overcomes these problems by treating the predictors as random variables and estimating them simultaneously. In the nonparametric kernel regressions (ELM and ULM), the numerous effects are estimable by smoothing the phenotypes against one predictor at a time, assuming that the effects of the remaining are removed from the phenotypes. Of course, the true effects of the remaining predictors are unknown and have to be estimated themselves, resulting in the iterative backfitting algorithm [5]. Nuisance factors can be included in the algorithm and can be estimated parametrically using least squares. The model is then semiparametric and the backfitting algorithm iterates between the parametric (*i.e*. estimating the effects of the nuisance factors by least squares) and the nonparametric part (*i.e*. estimating the SNP function values by the Nadaraya-Watson regression), without changing the general structure of the algorithm [5].

Using kernel regression, the choice of the appropriate degree of smoothing is important, which depends on the sample size. Naturally, if the sample size grows to infinity, smoothing is almost not required [7] and hence *λ *should be close to 1. However, sample size is never infinite, and, therefore, *λ *has to be chosen carefully, taking the sample size into account. Indeed, in ELM the optimal *λ *for a marker density of 1 cM, a heritability of 0.5 and applying the allelic model is 0.74 (Figure 1a). If the size of the data set would only be 500, the optimal *λ *would be 0.65 (not shown elsewhere). The applied bootstrap strategy takes the sample size into account, because the estimation set is of equal size as the full data set. In ELM the *λ *determined by bootstrapping was very close to the optimal *λ*. This can be seen by comparing the results reported in Table 2 for the ELM with the maximum achievable accuracies shown in Figures 1 and 2. Alternatively, leave-one-out cross validation is suggested [13,7]. Using this method, for a given *λ*, the functions are fitted using all but one observation and then the prediction error of this observation is calculated given the fitted functions. This is repeated for all observations. The *λ*, which produces the lowest average prediction error, is chosen to be the optimal *λ*. However, this strategy would require running *n *times the analysis, which would computationally be too demanding in the present data sets. The bootstrap as applied in this study is related to this cross-validation strategy, see [13] for a detailed discussion.

When nuisance factors are included in the model and the number of data points in some classes is very low, it might happen that in some bootstrap samples these effects are not estimable or estimated poorly. One obvious solution is to use only those bootstrap samples where the number of data points in each class is above a defined threshold. Since it is assumed that the nuisance effects and the SNP effects are independent, this would not affect the results regarding the choice of the appropriate *λ*.

From Figures 1 and 2 it can be seen that the regression coefficient was on average highest when the degree of smoothing was at maximum and decreased monotonically with a decrease of the degree of smoothing (higher *λ*), as expected. The crossing point of the regression plots with one (*i.e*. the unbiased estimation point) shown in the bottom of these figures coincided with the maximum accuracy (top of the figures). The plot of the accuracy against *λ *did not show a pronounced maximum. Hence, ELM was not very sensitive with regard to the choice of *λ*. The optimal *λ *depended on the marker density. With increasing density, more smoothing (*i.e*. a lower *λ*) was required. This is because the QTL effects are represented by all SNPs that are in LD with it. With an increasing number of SNP being in LD with the QTL, each SNP captures a smaller part of the QTL effect, and hence, requires more smoothing. Naturally, the number of SNP in LD with the QTL is higher in high marker density situations. Additionally, with increasing number of SNP, more SNP show by chance spurious effects, and hence, more smoothing is required to minimise the impact of these spurious effects. In this study the markers were equally distributed across the chromosomes. In practise it might happen that this is not the case and some QTL are in LD with many markers (requires more smoothing) whereas others only with few markers (requires less smoothing). It can be assumed that ULM might cope with unequal marker densities better than ELM and BLUP, because of the group-wise specific *λ *estimation.

The results from the allelic BLUP and the allelic ELM are very similar (Tables 1 and 2). This might be intuitively surprising, because of the different assumptions underlying these models. However, we compared both models formally and found close similarities between them, leading to the similar results. For details see Appendix 2. BLUP needs estimates of variance components whereas ELM needs a *λ*. For additive genetic variances reliable estimates of variance components are usual available, *e.g*. from REML analysis. However, this is in general not the case for nonadditive genetic variance components like dominance or epistasis. As reviewed by Thaller *et al*. [18], dominance QTL effects are not negligible. The nonparametric regression models allow the inclusion of dominance effects without having knowledge of the dominance variance component. A simulation study could show the benefit of taking dominance into account. However, for a realistic simulation knowledge of the distribution of QTL dominance effects is needed. This is largely unknown up to now. More research is needed in this field.

Meuwissen *et al*. [1] stated that the main disadvantage of BLUP is the assumption that every predictor is associated with the same genetic variance leading to a too strong regression of large QTL, which limits the accuracies of the *EBVs*. The same holds true for ELM, where the degree of smoothing is too strong for predictors linked to large QTL. ULM overcomes the problem of too strong smoothing of predictors with large QTL by building groups of *m *predictors showing similar variance of their function values and determining different *λ *for each group. Hence it is assumed that predictors that show a large variance are linked to large QTL. Indeed, in ULM the amount of smoothing is substantially reduced for many predictors (Tables 3 and 4), resulting in the higher accuracies of the *EBVs *estimated by ULM (Tables 1 and 2). The standard deviations in Tables 3 and 4 are high for *λ *> 0.7. This might be due to the difficulty in finding the optimal *λ *and additionally due to the unequal distribution of the simulated QTL effects. As described above, these followed gamma distribution with a high density for small and a low density for large effects [15]. Hence, some replicates might show several big QTL resulting in more predictors with a large *λ *whereas other replicates might show only small or medium sized QTL and the number of predictors with a *λ *close to one is small in these replicates as well.

In ULM a critical question is how large the group size (*m*) should be. If *m *is too small (*e.g. m *= 1 or 2) then only those predictors which are linked to very large QTL would receive a *λ *above that determined by ELM, because only these might be able to decrease the *aveRSS *during the grid search of *λ*. In contrast, if *m *is too large (*e.g. m *= 100 or 200), then many predictors containing only small QTL would receive a too large *λ*, because they are in a group with predictors with large QTL. Both situations would result in less accurate estimates. It seems that the group size chosen in this study (*m *in between 5 and 20, depending on the marker density) is an appropriate choice. The algorithm defining the group-wise *λ *was stopped when all predictors have passed it one time (see end of section 2.2). Alternatively the algorithm could have been repeated several times with updated *λ *and stopped when the *λ *did not change anymore, which would be, however, computationally very demanding.

It may be possible to estimate *λ *by the use of a prior distribution in ULM. One possibility for such a procedure would be to sample *λ *from a mixture of two distributions, one for predictors in LD with a QTL and the second component of the mixture for predictors not associated with a QTL. The latter distribution would put significantly more, if not all, probability mass at *λ *equal to 0.5 (smoothing is at maximum), whereas the first one would support less smoothing. However, as the models were implemented in this study, they do not use any prior information, in contrast to BayesB of Meuwissen *et al*. [1]. A comparison of the results presented in Table 2 with those of Solberg *et al*. [2], who simulated the same genetic configuration but applied BayesB, suggests that the accuracy of ULM is lower compared to the accuracies of BayesB in the allelic case.

## Conclusion

Nonparametric additive regression models for genomic breeding value estimation were shown to estimate breeding values of individuals without phenotypic information with moderate to high accuracy. The optimal degree of smoothing was determined either for all predictors jointly (ELM) or for groups of predictors separately (ULM). The latter increased the accuracies of the *EBVs*. The accuracies of the superior model, the ULM model, are in general slightly lower compared to BayesB. The behaviour of these models for the estimation of genomic breeding values considering also dominance QTL effects remains to be investigated.

## Competing interests

The authors declare that they have no competing interests.

## Authors' contributions

JB carried out the analysis and drafted the manuscript. TS simulated the data sets. TM helped to carry out the study and drafting the manuscript, and developed the Appendices. All authors have read and approved the final manuscript.

## Appendix 1

This appendix shows why the variance of the effects of each marker was assumed to be to be $\sigma_{a}^{2}$/(4*N*$\bar{H}$) in the BLUP model. $\sigma_{a}^{2}$ is the additive genetic variance, which is assumed to be known. *N *is the number of markers, $\bar{H}=\frac{1}{N}\sum_{i=1}^{N}2p_{i}(1-p_{i})$ is the average marker heterozygosity, and *p*_*i *_the allele frequency at marker *i*. Each marker M has two distinct alleles, M1 and M2, with effects *a*_*M*1 _and *a*_*M*2 _= (-*a*_*M*1_), respectively. The genotype frequencies are *p*^2^, 2*p*(1-*p*) and (1-*p*)^2^, respectively. The genetic mean is [5] (dropping the subscripts for ease of notation)

$$\begin{array}{l} \mu_{G}=p^{2}2a-{\left(1-p\right)}^{2}2a\\ =2a(2p-1). \end{array}$$

The sum of squares is

$$\begin{array}{l} SS=p^{2}4a^{2}+{\left(1-p\right)}^{2}4a^{2}\\ =-8a^{2}(p(1-p))+4a^{2}, \end{array}$$

and the variance explained by locus M is [5]

$$\begin{array}{l} \text{Var}(\text{M})=SS-\mu_{G}^{2}\\ =4a^{2}2p(1-p)\\ =4a^{2}H, \end{array}$$

where *H *is the heterozygosity at locus M. Assuming that $\sigma_{a}^{2}$ is equally distributed over all markers, var (M) is $\sigma_{a}^{2}$/*N*. Thus the expression above becomes

$$4a^{2}\bar{H}=\frac{\sigma_{\text{a}}^{\text{2}}}{N}.$$

The expectation of *a*^2 ^is the variance of a marker effect, *i.e. E*(*a*^2^) = var(*a*_*M*1_). In BLUP it is assumed that this is equal for all markers. Using this, the above expression becomes

$$4*\mathrm{var}(a_{M\text{1}})*\bar{H}=\frac{\sigma_{\text{a}}^{\text{2}}}{N}.$$

Hence, the variance of the marker effects is $\mathrm{var}(a_{M\text{1}})=\frac{\sigma_{\text{a}}^{\text{2}}}{\text{4}N\bar{H}}$, as used in this study, which is also in agreement with Habier *et al*. [14]. Note that if $\bar{H}$ = 0.5 (*i.e*. the allele frequency at each marker is 0.5) var(*a*_*M*1_) reduces to $\sigma_{a}^{2}$/(2*N*), which was used by Meuwissen *et al*. [1]. Note that each marker affects the phenotype two times, via the paternal and the maternal allele, hence the *2N *in the denominator (which is not mentioned in [1]).

## Appendix 2

This appendix shows the close similarity of the allelic BLUP model and the allelic nonparametric regression model using a single smoothing factor (*λ*) in eq (5) of the main text. A haploid model is assumed for simplicity of notation, but the extension to two alleles per marker is straightforward. Denote the number of times allele M1 (M2) at a marker M is observed in the sample as *n*_*M*1 _(*n*_*M*2_). The frequencies of M1 and M2 are *p *and 1-*p*. The mean of the phenotypes associated with M1 (M2) is ${\bar{y}}_{M1}({\bar{y}}_{M2})$. The sample mean is $\mu =p{\bar{y}}_{M1}+(1-p){\bar{y}}_{M2}$. Following mixed model theory, the BLUP prediction of a record with allele M1 (${\hat{u}}_{M1}$) is

(A1) $${\hat{u}}_{M1}=\mu +\frac{n_{M1}}{n_{M1}+k}\left({\bar{y}}_{M1}-\mu \right),$$

where *k *is the ratio of variances $\sigma_{e}^{2}$/($\sigma_{a}^{2}$/4*N*$\bar{H}$) (see appendix A). $\sigma_{e}^{2}$ is the error variance, $\sigma_{a}^{2}$ the additive genetic variance, and *N *the total number of markers. The term $\frac{n_{M1}}{n_{M1}+k}$ is denoted as *β*. Eq (A1) can be rearranged as

(A2) $$\begin{array}{lll} {\hat{u}}_{M1} & = & \beta ({\bar{y}}_{M1}-\mu )+\mu \\ {\hat{u}}_{M1} & = & \beta {\bar{y}}_{M1}+(1-\beta )\mu \\ & = & \beta {\bar{y}}_{M1}+(1-\beta )(p{\bar{y}}_{M1}+(1-p){\bar{y}}_{M2})\\ & = & (p+\beta (1-p)){\bar{y}}_{M1}+(1-p-\beta (1-p)){\bar{y}}_{M2}\\ & = & w_{1}{\bar{y}}_{M1}+w_{2}{\bar{y}}_{M2}, \end{array}$$

with *w*_1 _+ *w*_2 _= 1 and both weights are nonnegative. Hence, the BLUP estimate of the M1 effect is the weighted sum of the two means. The weights *w*_1 _and *w*_2 _depend on the variance component, *N*, gene frequencies, and *n*_*M*1 _and *n*_*M*2_. The BLUP estimate of the M2 effect can be derived in the same way.

According to eq (5) of the main text, the nonparametric function value of M1 can be written as

(A3) $$\hat{g}(\text{M}1)=\sum_{i=1}^{n}\sum_{k=1}^{2}v_{ik}y_{i},$$

with

(A4) $$v_{ik}=\frac{\lambda^{q-d(M1,x_{ik})}{\left(1-\lambda \right)}^{d(M1,x_{ik})}}{\sum_{i=1}^{n}\sum_{k=1}^{2}\lambda^{q-d(M1,x_{ik})}{\left(1-\lambda \right)}^{d(M1,x_{ik})}}.$$

As shown in the main text, in the allelic model *q *equals 2 and *d *can take the values 0 or 1, depending on the number of disagreements between the focal (M1) and the observed allele *x*_*ik *_and therefore *v*_*ik *_can take only two values, *v*_1 _(*v*_2_) for phenotypes associated with M1 (M2). Following this, (A3) results in

$$\hat{g}(\text{M}1)=v_{1}\sum_{i=1}^{n_{M1}}y_{M1,i}+v_{2}\sum_{i=1}^{n_{M2}}y_{M2,i},$$

where *y*_*M*1,*i *_and *y*_*M*2,*i *_denote for the phenotypes associated with M1 and M2, respectively. This can be written as

(A5) $$\begin{array}{c} \hat{g}(\text{M}1)=n_{M1}v_{1}\frac{1}{n_{M1}}\sum_{i=1}^{n_{M1}}y_{M1,i}+n_{M2}v_{2}\frac{1}{n_{M2}}\sum_{i=1}^{n_{M2}}y_{M2,i}\\ \begin{array}{l} =n_{M1}v_{1}{\bar{y}}_{M1}+n_{M2}v_{2}{\bar{y}}_{M2}\\ =w_{1}{\bar{y}}_{M1}+w_{2}{\bar{y}}_{M2}, \end{array} \end{array}$$

with *w*_1 _+ *w*_2 _= 1 and both weights are nonnegative. Here *w*_1 _and *w*_2 _depend on the degree of smoothing (*λ*) and on *n*_*M*1 _and *n*_*M*2_. The nonparametric function value of M2 can be expressed in the same way. Eq (A5) has the same form as (A2), hence by choosing *λ *appropriately, such that the weights *w*_1 _and *w*_2 _are similar or the same in BLUP and in the nonparametric regression, both models became similar or the same. If one *λ *is used across all loci, it becomes impossible to choose a *λ *such that the weights *w*_1 _and *w*_2 _are equal for both models for all loci. It may however be possible to choose *λ *such that these weights are very similar.
